# Malignant rhabdoid tumor of the liver in a middle-aged woman: a case report and literature review

**DOI:** 10.1186/s12876-022-02102-6

**Published:** 2022-01-21

**Authors:** Haikun Ye, Zirong Liu, Yamin Zhang

**Affiliations:** Hepatobiliary Surgery Department, Tianjin First Central Hospital, 24 Fukang Road, Nan Kai District, Tianjin, 300192 China

**Keywords:** Malignant rhabdoid tumor, Liver, SMARCB1, Case report

## Abstract

**Background:**

Extrarenal malignant rhabdoid tumor (EMRT) is a rare and high-mortality malignant tumor, which is more common in infants and rarely seen in adults. We firstly report a case of liver malignant rhabdoid tumor (MRT) with a loss of SMARCB1 gene (alias INI1, SNF5, BAF47) expression in a middle-aged woman, and preliminarily summarize the clinical characteristics and discuss its potential treatment of liver MRT by reviewing 55 cases reported in the past.

**Case presentation:**

We report a 40-year-old woman who was admitted to our hospital for right epigastric pain. Previously, the patient was treated with liver hematoma in another hospital until she came to our hospital for abdominal pain again. In our hospital, we performed surgical treatment on her and the pathology diagnosed EMRT with negative expression of SMARCB1. After surgery, the patient underwent genetic testing, but failed to screen for sensitive targeted or conventional chemotherapy drugs, and she did not receive further treatment. Due to lack of timely diagnosis and effective chemotherapy drugs, tumor recurrence and metastasis occurred one year after surgery. Then the patient chose traditional Chinese medicine for treatment. And the metastatic tumors had still progressed after one year of treatment, but the patient didn’t have obvious discomfort symptoms.

**Conclusions:**

Liver MRT is a highly aggressive tumor with high metastatic potential and poor prognosis. It lacks specific symptoms and signs and is easy to be ignored and misdiagnosed. The mortality rate is extremely high as there is no effective treatment. But most tumors are accompanied by SMARCB1 deficiency, which may offer new research directions for cancer therapeutics. For the present, early detection, early diagnosis and early resection remain the key to improve the prognosis of patients.

## Background

Malignant rhabdoid tumor (MRT) is a rare and highly aggressive tumor characterized by high metastatic risk, poor prognosis and high mortality. It’s most commonly encountered in the kidney, and has a low extrarenal incidence. Extrarenal malignant rhabdoid tumors (EMRTs) tend to occur in the body's central axis, mainly in infants and early childhood. Currently, only a few cases of adult primary liver MRT have been reported. In this paper, we describe a case of liver MRT in a 40-year-old woman for the first time.

## Case presentation

A 40-year-old woman was admitted to the hospital with the chief complaint of right upper abdominal pain for more than 4 months.

### Previous history and family history

The patient had no previous medical history or family history of tumors.

### Present history

**Fig. 1:**
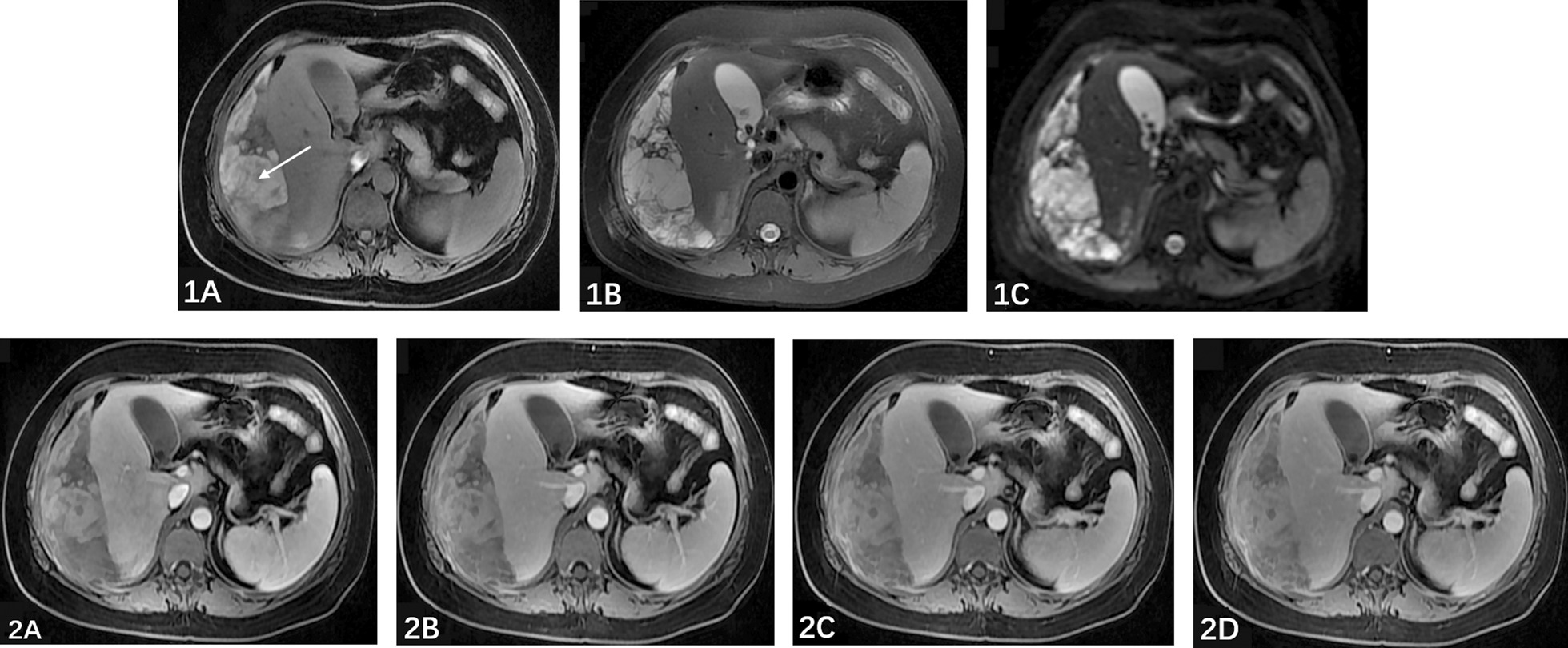
(2019-02-15) Abdominal MRI: an irregular mass with mixed short T1-weighted imaging (T1WI), mixed long T2-weighted imaging (T2WI) and high diffusion-weighted imaging (DWI) under the capsule of the right lobe of the liver. After enhancement, the mass showed uneven enhancement. (1A: T1WI sequence; 1B: T2WI sequence; 1C: DWI sequence; 2A: Arterial phase; 2B: Portal phase; 2C: venous phase; 2D: delayed phase.)

**Fig. 2:**
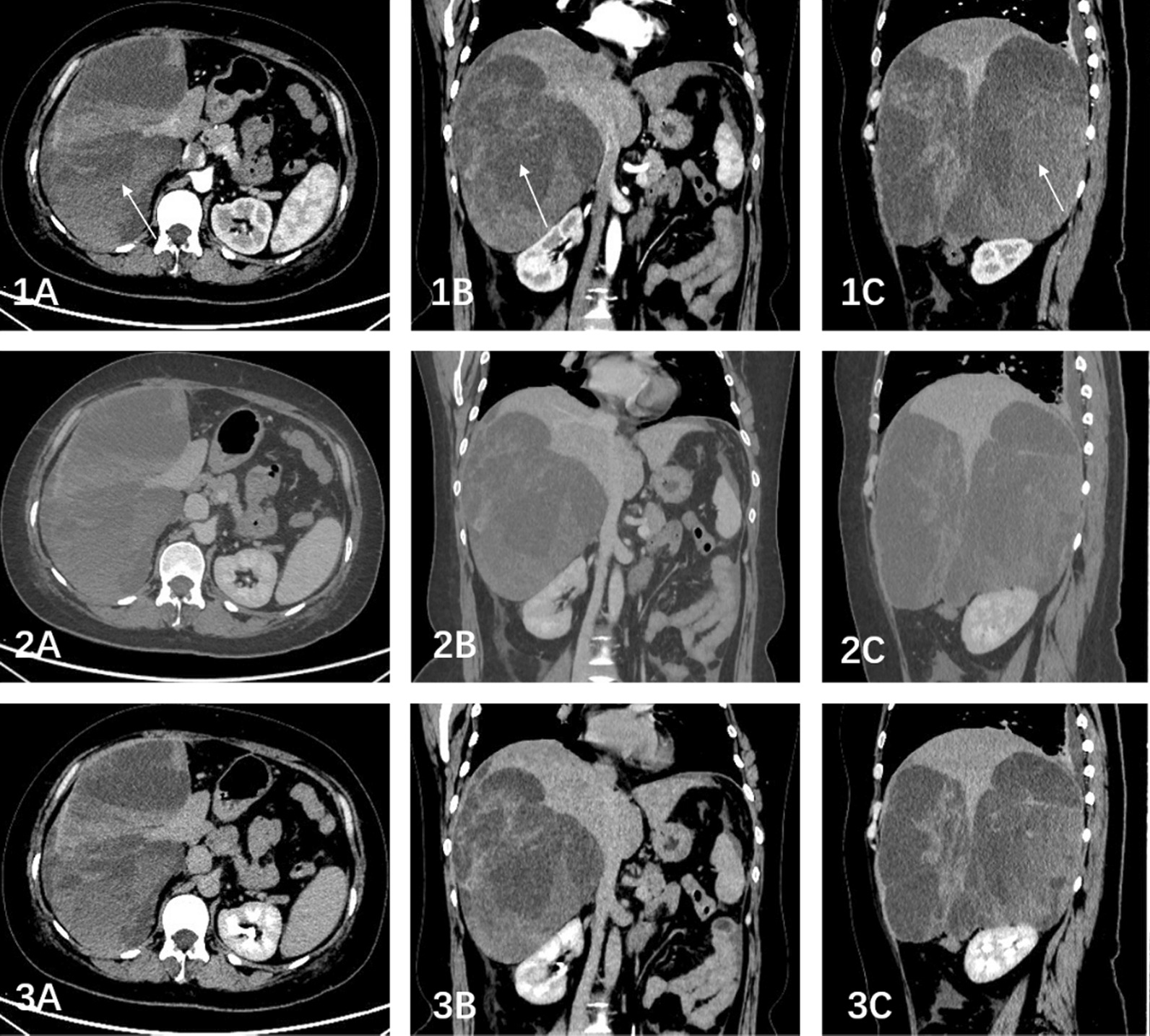
(2019-06-24) Abdominal enhanced-CT: multiple heterogeneous dense masses with regular morphology and clear boundaries between the right lobe of liver and the diaphragm. The largest one was about 13.4 × 9.2 cm and the CT value was between 20-45Hu with no obvious enhancement on enhanced scans. (1A: Axial arterial phase; 1B: Coronary arterial phase; 1C: Sagittal arterial phase; 2A: Axial portal phase; 2B: Coronary portal phase; 2C: Sagittal portal phase; 3A: Axial delayed phase; 3B: Coronary delayed phase; 3C: Sagittal delayed phase.)

More than 4 months before admission, the patient had a sudden outbreak of pain in the right upper abdomen without obvious inducement. The initial ultrasonography showed heterogeneous echo around the right liver, while computed tomography (CT) suggested subcapsular mass shadow with regular shape, clear boundary and uneven density, suggesting subcapsular haematoma. Magnetic resonance imaging (MRI) presented with an irregular mass with mixed short T1-weighted imaging (T1WI), mixed long T2-weighted imaging (T2WI) and high diffusion-weighted imaging (DWI) (Fig. 1). After conservative treatment in another hospital, the patient's symptoms were eliminated and the size of the tumor gradually decreased gradually as detected by regular MRI examination (every 1–2 months). However, the patient experienced acute right epigastric pain again six days before admission. Laboratory tests showed that haemoglobin was 111 g/L. Abdominal enhanced CT indicated multiple heterogeneous dense masses with regular morphology and clear boundaries between the right lobe of the liver and the diaphragm. The largest mass was approximately 13.4 × 9.2 cm and the CT value was between 20 and 45 Hu with no obvious enhancement of enhanced scans (Fig. 2). There was blood and fluid in the abdominal cavity. The right kidney was normal.

### Physical examination

The patient only had right upper abdominal tenderness and no other positive signs.

### Preoperative diagnosis

Liver-occupying lesion, HCC rupture hemorrhage?

### Operation

After admission, the patient was given conservative treatment such as hemostasis, analgesia and rehydration. Two days later, the hemoglobin level dropped to 82 g/L, with normal liver function, coagulation function, and tumor markers (AFP, CEA, and CA19-9). The tumor had a large amount of bleeding, but the patient's vital signs were stable. In this case, hepatic arterial embolization and then assessment of the cancer biology before surgery, or direct surgery were feasible treatment options. After communication with the patient and her family, they directly chose exploratory laparotomy. Intraoperatively, there was approximately 4000 ml old hemorrhage in the abdominal cavity. A large cystic mass with a size of 15 × 11 cm, was observed between the right hepatic lobe and diaphragm. There was no cirrhosis or obvious abnormalities in other abdominal tissues or organs. To ensure an adequate residual liver volume, we performed modified right hemihepatectomy (based on standard right hemihepatectomy, the liver cross-section was shifted approximately 1 cm to the right, and the middle hepatic vein was preserved).

### Pathological diagnosis

**Fig. 3:**
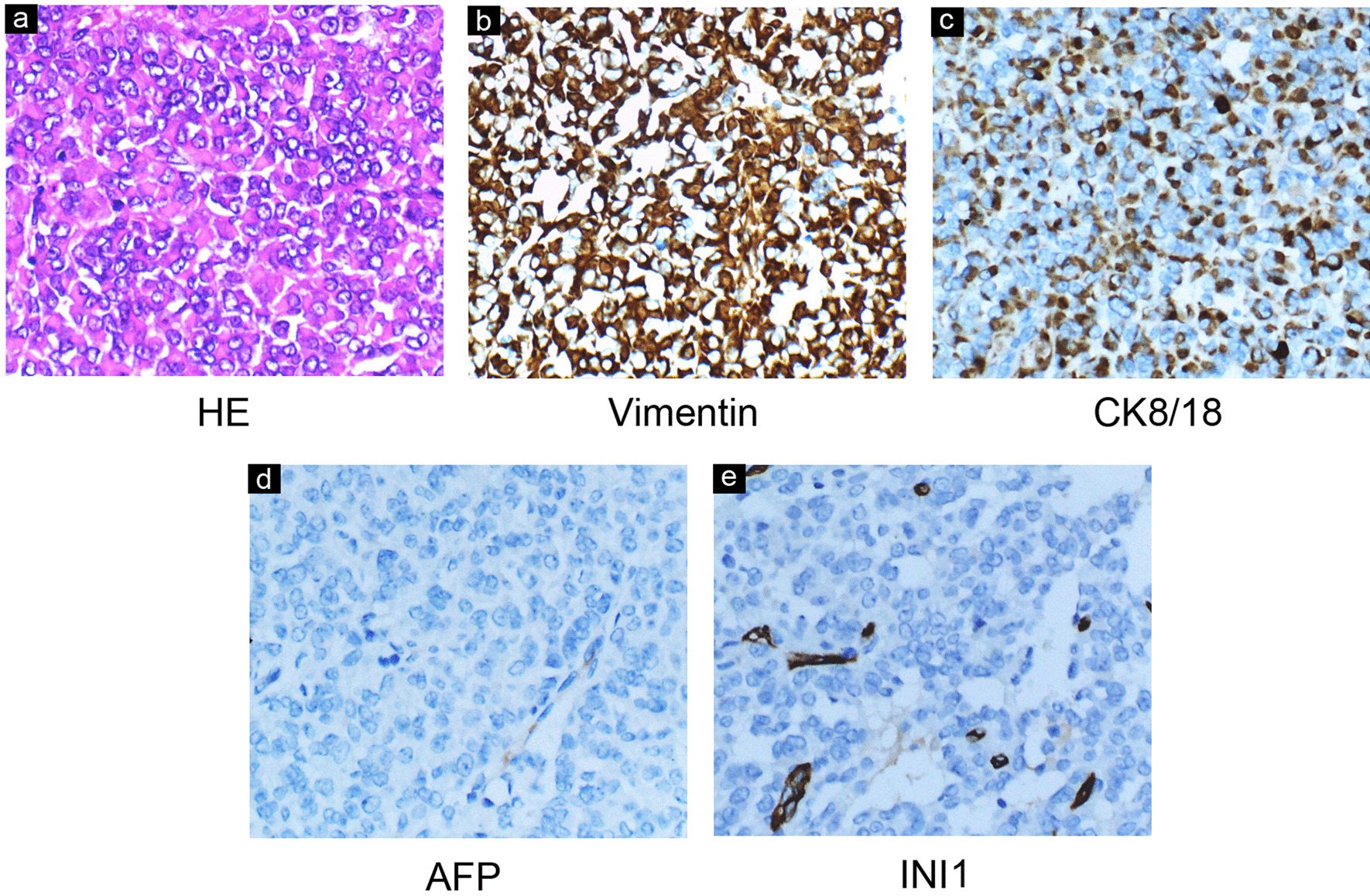
**a** Hematoxylin–eosin staining (HE): Pleomorphic tumor cells contain vesicular nuclei, with common mitotic phases, prominent nucleoli, and eosinophilic cytoplasm. Immunohistochemical stain showed diffuse cytoplasmic-positive staining for vimentin (**b**) and CK8/18 (**c**) and negative expression of AFP (**d**) and INI1 (**e**)

Postoperative pathology revealed pleomorphic tumor cells with vesicular nuclei, prominent nucleoli, and eosinophilic cytoplasm (Fig. 3a). Immunohistochemical staining showed diffuse cytoplasmic-positive staining for vimentin and cytokeratin (CK) 8/18 (Fig. 3b, c), focal positivity for CD56 and SMA, and negative expression of AFP (Fig. 3d). Further detection of INI-1 was also negative (Fig. 3e). The final pathological diagnosis was considered as EMRT.

### Postoperation

After surgery, the patient underwent genetic testing. Considering the patient's frail condition and while waiting for genetic testing results, the patient was discharged from hospital without immediate chemotherapy. Later, the results of genetic testing failed to screen out effective chemotherapy and targeted therapy drugs.

After discharge, the patient underwent abdominal CT examinations every 3–6 months, but did not receive further treatment. When abdominal CT in another hospital one year after surgery revealed metastatic tumors in the abdominal and pelvic cavity, the patient chose to be treated with traditional Chinese medicine (astragalus, scubelia, hedyotis, oyster and other medicinal materials). After one year of treatment with traditional Chinese medicine, based on what the patient described, abdominal CT reexamination showed that the metastatic tumors had progressed compared with before, but the patient was generally in good condition without obvious symptoms and signs.

## Discussion and conclusions

The histologic morphology of liver MRT, a rare subtype that portends a grim prognosis, is consistent with that of rhabdoid tumor in the kidney, and its histological origin is unclear [1]. Infants and young children are the main population of liver MRT. To date, a total of 55 cases of liver MRT have been reported on PubMed, including 51 pediatric patients and 4 adult patients, with a child-to-adult ratio of approximately 12:1. The median age of all pediatric patients was 7 months, and there was no sex difference. The 4 adult patients were male with ages of 27, 27, 50, and 51 [2–5] (Table 1). Liver MRT lacks specific symptoms and signs. Common symptoms and signs include fever, abdominal discomfort, abdominal mass, anorexia/vomiting and fatigue, with systemic symptoms predominating. Patients with severe symptoms are mostly associated with spontaneous rupture of the tumor.

**Table 1:** Liver MRT in adults

Author	Age	Sex	Symptoms	INI1	Laboratory tests	Imaging examinations	Treatment	Metastasis	Prognosis
Marzano [2] (2009)	27y	M	Acute epigastric pain	NR	AFP, CA19-9 normal	US: a large, heterogeneous mass	Left hepatectomy	NR	Alive after diagnosis 25 months
Sibileau [3] (2011)	27y	M	Asthenia, acute epigastric pain	Negative	Routine liver normal; AFP, CEA and CA19-9 normal	US: a heterogeneous left liver mass; CT: a voluminous low-density mass with edge enhancement; MRI: low signal on T1, heterogeneous high signal on T2	Left hepatectomy + chemotherapy	Without	Alive after diagnosis 41 months
Kang [4] (2013)	50y	M	Weight loss	Negative	AST/ALT/alkaline phosphatase elevate; AFP and CEA elevate, CA19-9 normal	CT: a very large multinodular hypoattenuating mass with rim enhancement	NR	NR	NR
Basir [5] (2017)	51y	M	Weight loss, dysphagia	Negative	AFP, CEA and CA19-9 normal	CT: hepatomegaly with multiple irregular hypoechoic necrotic lesions in both lobes	NR	Left adrenal widespread lymphadenopathies	NR

*M* male, *NR* not report, *AFP* alpha fetoprotein, *CEA* carcinoembryonic antigen, *CA19-9* carbohydrate antigen 19-9, *US* ultrasound, *CT* computed tomography, *MRI* magnetic resonance imaging

Imaging plays an indispensable role in disease diagnosis. Due to its rarity, there is no summative imaging feature of liver MRT. Reviewing the previous cases, liver MRT mostly manifests as solid or cystic masses with a heterogeneous echo by ultrasonography. CT usually exhibits single or multiple heterogeneous low-density masses, while the tumor exhibits heterogeneous enhancement after enhancement, possibly accompanied by calcification, necrosis and hemorrhage. The MRI findings have been described as hypointense on T1WI and heterogeneous hyperintense on T2WI, with mild enhancement or peripheral enhancement during enhancement.

The diagnosis of liver MRT still relies on the pathology. Microscopically, the tumor cells are round or polygonal, and composed of frequent mitosis, prominent nucleoli and abundant eosinophilic cytoplasm [6]. In immunohistochemical analysis, most tumors show high expression of vimentin, cytokeratin and smooth muscle actin (SMA), but negative expression of AFP, CD34 and myoglobin [6, 7]. However, several tumors, such as rhabdomyosarcoma, rhabdoid melanoma and epithelioid sarcoma, are similar to MRT in histopathology [8]. Thus, if only relying on microscopic cell morphology and common immunohistochemical markers, the final diagnosis must be established by carefully excluding other similar tumors. If possible, the expression of INI1 can be detected, which is of great value in the diagnosis of MRT. In the past, all 31 cases tested for INI1 were negative.

Currently, there are still no standard treatments for liver MRT. In previous cases, surgery, chemotherapy, radiotherapy and combinations of the three methods were used, and nearly all patients were treated with commonly used chemotherapy drugs such as vincristine, doxorubicin, etoposide, cyclophosphamide, carboplatin, cisplatin and ifosfamide. Although multimodal therapies were adopted, the survival time of most patients was very short; 82% of patients died within 6 months and 94% died within 1 year of diagnosis. The targeted drug bevacizumab has also been used to treat MRT but ended in failure [9]. One patient survived more than 3 years after liver transplantation and chemotherapy [10]. For those satisfying the criteria, liver transplantation may be an alternative approach. Nevertheless, up to 58% of patients displayed metastases at their first visit, and the application of liver transplantation is extremely limited.

To the best of our knowledge, this is the first report of liver MRT in an adult female, mainly manifesting as rupture and hemorrhage. The imaging and pathological findings of the patient were basically consistent with those that were previously reported. Unfortunately, one year after surgery, the patient developed abdominal and pelvic metastasis, suggesting that the tumor was highly aggressive. Moreover, gene detection failed to screen for sensitive chemotherapeutic drugs. Therefore, there is an urgent need to develop an effective treatment for liver MRT.

**Fig. 4:**
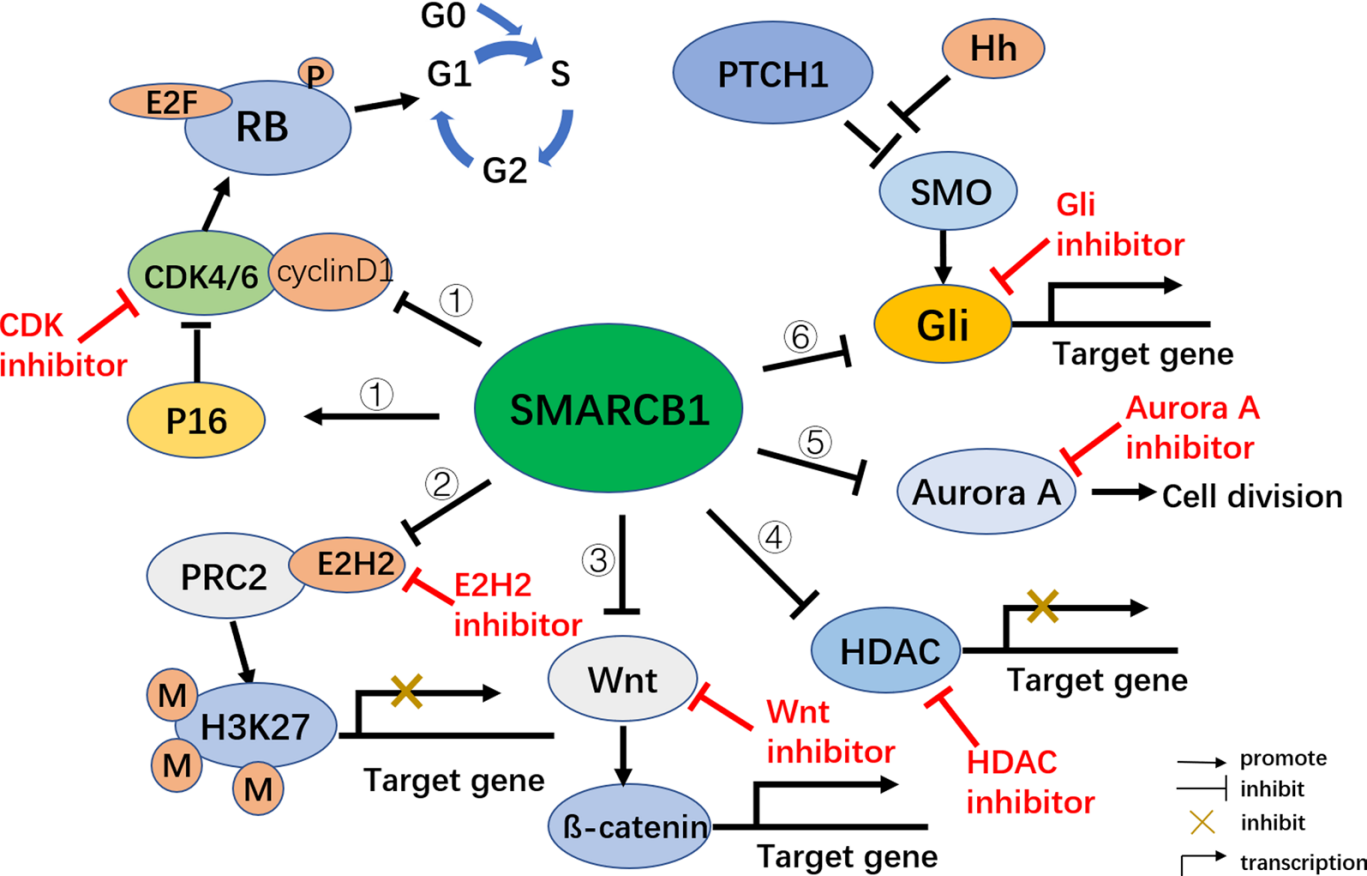
SMARCB1-related signal transduction and potential therapeutic targets (Red). ①: SMARCB1 negatively regulates the transformation of tumor cells from G0/G1 phase to S phase by regulating P16/RB pathway. ②: SMARCB1 negatively regulates the trimethylation of H3K27 by inhibiting the expression of EZH2, thus regulating the expression of target genes. ③: SMARCB1 inhibits the expression of target genes by regulating the Wnt/ß-catenin pathway, thus changing the cell phenotype. ④: SMARCB1 regulates the expression of target genes by inhibiting HDAC expression. ⑤: SMARCB1 inhibits tumor cell division by inhibiting Aurora A expression. ⑥: Hh releases the inhibition of SMO protein activity by PTCH1, thereby promoting the nuclear accumulation of downstream Gli protein and the transcription of downstream target genes. SMARCB1 negatively regulates downstream target genes by inhibiting GLI expression.)

Genetic and epigenetic alterations play crucial roles in the initiation and progression of tumors. As a product of genetic and epigenetic dysregulation, gene and epigenetic therapy may provide new directions for MRT treatment. Deletion of the SMARCB1 locus (alias INI1, SNF5, BAF47) in chromosome 22q11.2 is the most specific change in MRT [11]. SMARCB1 encodes the core subunit of the SWI/SNF chromatin remodelling complex, which can directly recruit histone deacetylase activity to the cyclin D1 promoter and mediate cell-cycle arrest, and is presumed to function as a tumor suppressor [12]. Exogenous introduction of the SMARCB1 gene may restore its function for biallelic inactivation of SMARCB1. Simultaneously, loss of SMARCB1 function can activate Wnt/catenin, PI3K/Akt, hedgehog(Hh)/GLI, PRC2/H3K27 and other signalling pathways, promote the high expression of HDAC and Aurora A, and lead to changes in epigenetic regulation of the cell cycle, proliferation and differentiation (Fig. 4) [13, 14]. In addition, loss of SMARCB1 function contributes to transcriptional activation of the antiapoptotic protein MCL-1, inhibits the proapoptotic protein Noxa and induces resistance of MRT cells to topoisomerase II inhibitors (such as doxorubicin, etoposide and doxorubicin) [15]. Thus, regulation of these pathways may also play a role in the treatment of MRT.

Several inhibitors of histone modifiers have been shown to sensitize MRT cells to radiotherapy and chemotherapy, such as enhancer of zeste homolog 2 (EZH2) and histone deacetylase (HDAC) [16]. The MCL-1 inhibitor TW-37 was also shown to enhance the ability of doxorubicin to induce MRT cell death [15]. Additionally, loss of SMARCB1 is accompanied by the upregulation of cyclin D1 and cyclin-dependent kinases (CDKs) [17]. All of these factors may become potential targets for the clinical treatment of MRT in the future.

Notably, approximately 5% of MRTs do not have SMARCB1 deletions but lack SMARCA4 gene expression, and its function is still unclear [18]. The absence of SMARCB1 occurs not only in MRT, but also in some nonrhabdomyoid tumors [19]. Therefore, we need to perform an increasing number of in-depth studies to further explore the occurrence and development mechanism of MRT.

In conclusion, liver MRT is a rare tumor but also one of the most lethal malignancies. Despite the existence of intensive multimodal therapy currently, the curative effect is fairly dismal. The SMARCB1 deficiency, along with the changes in various signaling pathways, may offer new research directions for cancer therapeutics. Nevertheless, the mechanisms of the occurrence and development of liver MRT are still unclear, and we are obliged to carry out more in-depth researches to elucidate it. With the progress of medicine, we have faith that more patients will receive efficacious treatment and better prognosis.

## Data Availability

All data are included in this current article.

## Abbreviations

EMRT: Extrarenal malignant rhabdoid tumor
MRT: Malignant rhabdoid tumor
INI1: Integrase interactor 1
SNF5: Sucrose nonfermentable 5
BAF47: BRG1-Associated Factor 47
SMARCB1: SWI/SNF-related matrix-associated actin-dependent regulator of chromatin subfamily B member 1
CT: Computed tomography
MRI: Magnetic resonance imaging
T1WI: T1-weighted imaging
T2WI: T2-weighted imaging
DWI: Diffusion-weighted imaging
HCC: Hepatocellular carcinoma
AFP: Alpha-fetoprotein
CEA: Carcinoembryonic antigen
CA19-9: Carbohydrate antigen 19-9
CK: Cytokeratin
CD56: Cluster of Differentiation 56
SMA: Smooth muscle actin
CD34: Cluster of Differentiation 34
PI3K: Phosphatidylinositol 3 kinase
Akt: Protein kinase B
Hh: Hedgehog
PRC2: Polycombrepressive complex 2
GLI: Glioma-associated oncogene homolog
H3K27: Histone 3 lysine 27
MCL-1: Myeloid cell leukemia-1
EZH2: Enhancer of zeste homolog 2
HDAC: Histone deacetylase
CDKs: Cyclin-dependent kinases
SMARCA4: SWI/SNF-related matrix-associated actin-dependent regulator of chromatin subfamily A member 4
